# Supplementary material for: Independent and joint associations of volume and intensity of physical activity on cognitive impairment among middle-aged and elderly Chinese adults: A national longitudinal study
Source: PLoS One. 2025 Dec 30;20(12):e0339124. doi: 10.1371/journal.pone.0339124 (PMC12752972; doi:10.1371/journal.pone.0339124)
Supplement: S1 Table — (DOCX) [file pone.0339124.s001.docx]

S1 Table. Association between PA (volume and intensity) and the risk of cognitive impairment

| PAV | Insufficient PA | 600-1199 MET-min/week | 1200-1799 MET-min/week | 1800-2999 MET-min/week | 3000-5999 MET-min/week | 6000-8999 MET-min/week | ≥9000 MET-min/week |
| --- | --- | --- | --- | --- | --- | --- | --- |
| Age |  |  |  |  |  |  |  |
| ＜65 | Ref | 0.708(0.473-1.058) | 0.866(0.677-1.107) | 0.645(0.478-0.869) * | 0.891(0.734-1.081) | 0.874(0.712-1.074) | 1.058(0.899-1.244) |
| ≥65 | Ref | 0.783(0.499-1.231) | 0.751(0.585-0.963) * | 0.641(0.448-0.918) * | 0.890(0.713-1.110) | 0.739(0.567-0.962) * | 0.853(0.693-1.049) |
| Gender |  |  |  |  |  |  |  |
| Men | Ref | 0.874(0.537-1.423) | 0.790(0.579-1.079) | 0.503(0.319-0.794) * | 0.745(0.572-0.971) * | 0.689(0.516-0.921) * | 0.753(0.604-0.940) * |
| Women | Ref | 0.658(0.449-0.965) * | 0.818(0.662-1.011) | 0.694(0.533-0.905) * | 0.942(0.792-1.121) | 0.864(0.712-1.049) | 1.098(0.943-1.277) |
| Residence |  |  |  |  |  |  |  |
| Rural | Ref | 0.782(0.573-1.068) | 0.849(0.701-1.028) | 0.625(0.487-0.802) * | 0.886(0.758-1.035) | 0.812(0.686-0.962) * | 0.982(0.862-1.120) |
| Urban or other | Ref | 0.345(0.107-1.111) | 0.632(0.409-0.976) * | 0.670(0.373-1.203) | 0.782(0.520-1.176) | 0.815(0.485-1.370) | 0.874(0.532-1.435) |
| IPA |  | Proportion of LPA to PAV | | | Proportion of MVPA to PAV | | |
|  | 0-0.25 | 0.25-0.5 | 0.5-0.75 | 0.75-1 | 0.25-0.5 | 0.5-0.75 | 0.75-1 |
| Age |  |  |  |  |  |  |  |
| ＜65 | Ref | 0.924(0.804-1.063) | 0.867(0.677-1.111) | 0.970(0.842-1.117) | 0.857(0.663-1.106) | 0.913(0.783-1.065) | 0.965(0.848-1.098) |
| ≥65 | Ref | 0.746(0.610-0.912) * | 0.538(0.370-0.782) * | 0.832(0.702-0.986) * | 0.588(0.405-0.855) * | 0.815(0.667-0.997) * | 1.029(0.867-1.222) |
| Gender |  |  |  |  |  |  |  |
| Men | Ref | 0.882(0.716-1.087) | 0.670(0.446-1.006) | 1.149(0.951-1.388) | 0.551(0.365-0.832) * | 0.725(0.582-0.904) * | 0.760(0.636-0.908) * |
| Women | Ref | 0.852(0.743-0.977) * | 0.773(0.608-0.982) * | 0.832(0.729-0.951) * | 0.861(0.674-1.099) | 0.949(0.821-1.097) | 1.093(0.964-1.240) |
| Residence |  |  |  |  |  |  |  |
| Rural | Ref | 0.900(0.799-1.013) | 0.789(0.637-0.978) * | 0.948(0.845-1.063) | 0.790(0.635-0.983) * | 0.900(0.792-1.023) | 0.970(0.872-1.081) |
| Urban or other | Ref | 0.513(0.337-0.779) * | 0.412(0.189-0.895) * | 0.651(0.471-0.900) * | 0.537(0.249-1.154) | 0.665(0.445-0.994) * | 1.117(0.777-1.606) |

PA - physical activity; PAV - Physical activity volume; MET-min/week - metabolic equivalents-minutes /week; MVPA - moderate to vigorous physical activity; LPA - low-intensity physical activity; Ref - reference. Adjusted for age, gender, smoking status, alcohol consumption, education, BMI chronic disease conditions except the subgroup variable itself. * P < 0.05.
