# Supplementary material for: Independent and joint associations of volume and intensity of physical activity on cognitive impairment among middle-aged and elderly Chinese adults: A national longitudinal study
Source: PLoS One. 2025 Dec 30;20(12):e0339124. doi: 10.1371/journal.pone.0339124 (PMC12752972; doi:10.1371/journal.pone.0339124)
Supplement: Attachment — Submitted filename: PONE-D25-38561 REVIEW.docx [file pone.0339124.s002.docx]

**PONE-D -25-38651 REVIEW**

- Thank you for the opportunity to review the manuscript entitled “Independent and joint associations of volume and intensity of physical activity on cognitive impairment among middle-aged and elderly Chinese adults: a national longitudinal study”
- The authors essentially measured physical activity and risk for cognitive impairment. They compared participants in the following manner: Baseline characteristics were compared between groups with insufficient vs. sufficient physical activity (PA), using percentages and chi-square tests for categorical variables. They then did a risk analysis using Cox proportional hazards models. Hazard ratios (HRs) were calculated using 95% confidence intervals to assess the association between PA volume/intensity and cognitive impairment risk.
- Model Adjustments were done and presented as model I: Adjusted for age and gender, model II: adding smoking, alcohol use, education, and BMI and a final model III, which Included all study covariates.
- Joint effects were measured /done by means of Restricted Cubic Splines (RCS), which evaluate d the combined effect of moderate-to-vigorous PA (MVPA) and light PA (LPA) on cognitive impairment risk. Participants were the grouped based on MVPA and LPA proportions.
- The subgroup analyses were then conducted separately by: Age (<65 vs. ≥65 years), Gender (male vs. female) and residence (rural vs. urban).
- Statistical analyses were performed using STATA 14 and R, with significance set at p < 0.05.
- Based on this, it seems regardless of how much total physical activity someone does, the mix of intensity matters, with balanced activity — with moderate MVPA (25–50%) and high LPA (50–75%) — linked to the lowest risk of cognitive impairment. Low proportions of either MVPA or LPA are associated with higher risk, even if overall activity levels are high. I suggest the following be highlighted clearly: the importance of diversifying activity intensity, not just increasing total volume.
- Presentation of the results is sound, with the forest plot visually supporting the quantitative relationships described in the text. Both indicate that balanced physical activity — with moderate MVPA and higher LPA — is associated with lower cognitive impairment risk, regardless of total activity volume.
- Regardless of the above summary, in general, I found the manuscript of good quality (some comments for minor edits made re grammar, see attached) and I commend the authors for their original contribution to a very important and relevant area of study in neuropsychiatry. In addition, the cross-cutting issues for exercise science disciplines provides additional value.

The longitudinal nature of the study, as well as big sample size add further add merit.

- **A few things for noting:**

I suggest that the term “cognitive impairment” be clearly commented on right at the outset. It must be noted that cognitive decline with aging process may be mild, moderate or severe, with classifications for neurodegenerative disorders such as Dementia are generally delineated into minor or major neurocognitive disorders of which Alzheimer’s dementia is an example. From the outset, it would be best to define what exactly this study is examining- cognitive impairment or dementia? In line 87 it appears as if the study refers to neurodegenerative disorders in general.

In the methodology, please indicate clearly (perhaps add to the flow chart on page 5) when exactly were the physical activity measures undertaken (i.e. when was the IPAQ administered?)
