# Supplementary material for: Independent and joint associations of volume and intensity of physical activity on cognitive impairment among middle-aged and elderly Chinese adults: A national longitudinal study
Source: PLoS One. 2025 Dec 30;20(12):e0339124. doi: 10.1371/journal.pone.0339124 (PMC12752972; doi:10.1371/journal.pone.0339124)
Supplement: Attachment — Submitted filename: Response to Reviewers.docx [file pone.0339124.s003.docx]

*Response by Wei Yin and Hongmei Sun et al.*

*Ref: PONE-D-25-38651*

*Title: Independent and joint associations of volume and intensity of physical activity on cognitive impairment among middle-aged and elderly Chinese adults: a national longitudinal study*

*PLOS ONE

Dear Dr. Sun,

Thank you for submitting your manuscript to PLOS ONE. After careful consideration, we feel that it has merit but does not fully meet PLOS ONE’s publication criteria as it currently stands. Therefore, we invite you to submit a revised version of the manuscript that addresses the points raised during the review process.

Please submit your revised manuscript by Dec 12 2025 11:59PM. If you will need more time than this to complete your revisions, please reply to this message or contact the journal office at* [*plosone@plos.org*](mailto:plosone@plos.org)*. When you're ready to submit your revision, log on to https://www.editorialmanager.com/pone/ and select the 'Submissions Needing Revision' folder to locate your manuscript file.

Please include the following items when submitting your revised manuscript:*

- *A rebuttal letter that responds to each point raised by the academic editor and reviewer(s). You should upload this letter as a separate file labeled 'Response to Reviewers'.*
- *A marked-up copy of your manuscript that highlights changes made to the original version. You should upload this as a separate file labeled 'Revised Manuscript with Track Changes'.*
- *An unmarked version of your revised paper without tracked changes. You should upload this as a separate file labeled 'Manuscript'.*

*If you would like to make changes to your financial disclosure, please include your updated statement in your cover letter. Guidelines for resubmitting your figure files are available below the reviewer comments at the end of this letter.

If applicable, we recommend that you deposit your laboratory protocols in protocols.io to enhance the reproducibility of your results. Protocols.io assigns your protocol its own identifier (DOI) so that it can be cited independently in the future. For instructions see:* [*https://journals.plos.org/plosone/s/submission-guidelines#loc-laboratory-protocols*](https://track.editorialmanager.com/CL0/https:%2F%2Fjournals.plos.org%2Fplosone%2Fs%2Fsubmission-guidelines%23loc-laboratory-protocols/1/010f019a29afaf9d-a7e5ef10-a655-4312-9bdf-934bf6a93f32-000000/no4K6nHDnX3bqokgzpmoopCXr_rwQKyTWYBHuMWCraM=234)*. Additionally, PLOS ONE offers an option for publishing peer-reviewed Lab Protocol articles, which describe protocols hosted on protocols.io. Read more information on sharing protocols at* [*https://plos.org/protocols?utm_medium=editorial-email&utm_source=authorletters&utm_campaign=protocols*](https://track.editorialmanager.com/CL0/https:%2F%2Fplos.org%2Fprotocols%3Futm_medium=editorial-email%26utm_source=authorletters%26utm_campaign=protocols/1/010f019a29afaf9d-a7e5ef10-a655-4312-9bdf-934bf6a93f32-000000/G0NnRbEgrp3O98V6yybPALxr0su4eLmHzjCKadRiHmQ=234)*.

We look forward to receiving your revised manuscript.*

*Kind regards,*

*Demitri Constantinou, MD
Academic Editor
PLOS ONE*

*HANDLING EDITOR*

***Comments***

*Thank you for submitting your manuscript to PLOS ONE. After careful consideration, we feel that it has merit but does not fully meet PLOS ONE’s publication criteria as it currently stands. Therefore, we invite you to submit a revised version of the manuscript that addresses the points raised during the review process.*

**Response:** Many thanks to the editor for your kind words and the chance for revision submission. We have carefully revised it in accordance with all the reviewers’ comments. Below, we provide point-by-point responses to their feedback. The reviewers’ comments are displayed in blue, our responses in black, and additions to the revised manuscript are highlighted in red.

*Reviewer #1: PLOS ONE*

*Ref: PONE-D-25-38651*

*Title: “Independent and joint associations of volume and intensity of physical activity on cognitive impairment among middle-aged and elderly Chinese adults: a national longitudinal study”*

This is an interesting paper on physical activity and cognitive impairment in middle and older adults in China. It is a well-organized paper with a good literature review, statistical rigor, and discussion.
**Response:** We sincerely thank the reviewer for the insightful and encouraging comments. We truly appreciate your recognition of the novelty and public health significance of our study. We have carefully addressed each of your suggestions and have revised the manuscript to improve the clarity of writing and ensure overall structural completeness. Detailed point-by-point responses are provided below. We hope the revised manuscript and our responses satisfactorily address your concerns and meet your expectations.

Comment 1: A general comment would be that there are a lot of abbreviations within the abstract. It’d be good if you could summarize those to make it more interesting to read, especially the results part.
**Response:** We thank the reviewer for this valuable comment. We have summarized abbreviations to make it more interesting to read within the abstract. Then, we have provided the full name for each abbreviation upon its first appearance. **(Abstracts section, page 2, lines 36-56)**.

**The revised paragraph now reads as follows:**

**Abstracts**

**Background:** The objective of this study was to explore the longitudinal relationship between the volume and intensity of **physical activity (PA) and their combined risk for cognitive impairment (****CI).**

**Methods:** The study included 10,174 participants from the 2011-2018 CHARLS cohort. PA and CI were assessed using self-reported questionnaires. Statistical analyses were performed using the **Cox regression model.**

**Results:** After adjusting for all covariates, the risk of CI was 14% lower in subjects with **physical activity volume (PAV)** ≥600 **Metabolic Equivalent of Task (MET)**-min/week compared to those with insufficient PA (HR: 0.86). The risk was reduced by 38% for subjects with PAV of 1800-2999 MET-min/week (HR: 0.62). Regarding the intensity of PA, the risk of CI was reduced by 25% for a proportion of 0.25-0.5 of **(moderate to vigorous PA) MVPA** to PAV (HR: 0.75) compared to a proportion of 0-0.25. **Regardless of PAV, the risk of CI was lowest when the proportion of moderate to vigorous PA(MVPA) to PAV was 0.25-0.5, and 0.5-0.75 for the proportion of light-intensity physical activity (LPA) to PAV.**

**Conclusion:** The PAV 1800-2999 MET-min/week and maintaining a proportion of LPA to PAV of 0.5-0.75, or a proportion of MVPA to PAV of 0.25-0.5, are more effective in reducing the risk of CI. Policy implications should prioritize tailored physical activity strategies for individuals over 65, emphasizing low-intensity activities, safe high-intensity training, and the development of accessible urban facilities, in line with WHO guidelines.

Comment 2: In the literature review, lines 95-96 cite an article about machine learning and depression, not sure how that is relevant to the sentence there. Please revise that.
**Response:** We appreciate this suggestion. We have removed the article about machine learning and depression in the literature review, and we have added the new references. **(Introduction section, page 4, lines 95-97)**.

**The revised paragraph now reads as follows:**

“Introduction

…Survey To the best of our knowledge, specific guidelines for PA to prevent the onset of cognitive impairment remain absent. Furthermore, the causal relationship between PA and cognitive functioning is still a subject of debate in the existing research. **One study observed engaging in high-intensity PA may lead to cognitive decline in middle-aged and older adults [20]**, whereas another study found that moderate-to-high intensity aerobic physical activity did not slow cognitive impairment in people with mild-to-moderate dementia [21]. …”

**The relevant certificate involved is listed as follows:**

20.	Temesgen, W.A., H.Y. Cheng, and Y.Y. Chong, Cognitive function and its longitudinal predictability by intensity of physical activity in Chinese middle-aged and older adults. J Alzheimers Dis, 2025. 103(3): p. 809-820.

Comment 3: Line 125, there is an abbreviation “ID” without a previous explanation. Please review that.

**Response:** Thanks for your valuable advice. We have revised the abbreviation “ID” to “identity document”. Thanks. **(Methods Materials and methods section, page 5, lines 127)**.

**The revised paragraph now reads as follows:**

“Methods Materials and methods

Participants and study design

…

Survey data from 2011-2018 were combined by **identity document**‌, resulting in a longitudinal survey of 13,263 data. There were 489 data with positive cognitive impairment at baseline. …”

Comment 4: In lines 172-182, phrases like ‘such as’, ‘including…’ are used, and this might indicate there are more variables listed below, so rephrase it (unless there are more variables not mentioned in that paragraph)

**Response:** Thanks for your valuable advice. We have deleted those (‘such as’, ‘including…’) in the manuscript. **(Methods Materials and methods section, page 7, lines 175-184)**.

**The revised paragraph now reads as follows:**

“Methods Materials and methods

Covariates

…

According to previous articles [27, 34]，covariates are primarily categorized into the following groups: 1) Personal variables: age, gender (male, female), education level (high school or below, undergraduate or higher), and residence (rural, urban, or other); 2) Health status-related variables: body mass index (BMI) are categorized as follows: underweight (<18.5), normal weight (18.5 ≤ BMI <25), overweight (25 ≤ BMI <30), and obese (BMI ≥30); 3) Lifestyle variables, smoking status (current smoker, former smoker, never smoker) and alcohol consumption (current drinker, former drinker, never drinker); 4) Chronic disease conditions: hypertension (no, yes), hyperlipidemia (no, yes), diabetes (no, yes), and lung disease (no, yes).”

*Reviewer #2: PLOS ONE*

*Ref: PONE-D-25-38651*

*Title: “Independent and joint associations of volume and intensity of physical activity on cognitive impairment among middle-aged and elderly Chinese adults: a national longitudinal study”*

In general, the language is good. however, there are some areas for suggested improvement.

**Response:** We thank for your valuable comments and suggestions. We have carefully revised the manuscript to enhance its clarity and facilitate the understanding of readers. Our point-to-point responses are presented in the following.

minor edits only:

Comment 1: in the abstract: Suggest "....and their combined risk for cognitive impairment", rather than "their combination"
**Response:** We thank the reviewer for this valuable comment. We have revised " their combined " to "their combination". **(Abstracts section, page 2, lines 37-38)**.

**The revised paragraph now reads as follows:**

“Abstracts

Background: The objective of this study was to explore the longitudinal relationship between the volume and intensity of physical activity (PA) and **their combined** risk for cognitive impairment (CI).”

Comment 2: In the introduction, a suggestion to define cognitive impairment in the context of the study from the outset. Define what exactly this study is examining- cognitive impairment or dementia (which is a major neurocognitive disorder) cognitive impairment should ideally be delineated in terms of mild, moderate or severe, which dementia is an example of. NB in line 87 the study appears to examine neurodegenerative disorders.

**Response:** We sincerely thank the reviewer for the valuable suggestion. We have added define cognitive impairment in the context of the study in the introduction. In addition, we have deleted the study appears to examine neurodegenerative disorders in line 87. **(Introduction section, page 3, lines 65-67)**.

**The revised paragraph now reads as follows:**

“Introduction

Cognitive impairment (CI) refers to a significant decline or dysfunction in memory, learning, attention, language, executive function, and other cognitive domains[1], ^…”^

**The relevant certificate involved is listed as follows:**

1.	Petersen, R.C., et al., Mild cognitive impairment: ten years later. Arch Neurol, 2009. 66(12): p. 1447-55.

Comment 3: line 79, " The World Health Organization (WHO) recommends 150-300 minutes of moderate-intensity physical activity per week, 75-150 minutes of vigorous-intensity physical activity, or some equivalent combination of moderate intensity and vigorous-intensity aerobic physical activity per week. " suggest the authors indicate what exactly for? to maintain a good physique/remain healthy/ avoid dementia, prevent various illnesses?
**Response:** We thank the reviewer for this insightful suggestion. We have revised "The World Health Organization (WHO) recommends 150-300 minutes of moderate-intensity physical activity per week, 75-150 minutes of vigorous-intensity physical activity, or some equivalent combination of moderate intensity and vigorous-intensity aerobic physical activity per week." to "The World Health Organization (WHO) recommends 150-300 minutes of moderate-intensity physical activity per week, 75-150 minutes of vigorous-intensity physical activity, or some equivalent combination of moderate-intensity and vigorous-intensity aerobic physical activity per week to gain health benefits such as improvements of cognitive health".**(** **Introduction section, page 3, lines 82-85)**.

Comment 4: line 106-108: check grammar. suggest "utilized" or motivate for the study adequately.

**Response:** We sincerely thank for the valuable comment. We have revised to "utilized". **(Introduction section, page 4, lines 108)**.

**The revised paragraph now reads as follows:**

“Introduction

…Based on this, this study **utilized** a nationally representative sample from the China Health and Retirement Longitudinal Study (CHARLS) to explore the longitudinal relationship between volume and intensity of PA and their combined risk for CI.”

Comment 5: line 126-129: suggest full stop after the word "baseline" in "There were 489 data with positive cognitive impairment at 127 baseline. then: "Twenty five with missing data in PA and cognitive impairment data at baseline were excluded, and 1487 with missing data in longitudinal cognitive impairment data were excluded, leaving 11262 data."

**Response:** We sincerely thank for the valuable comment. We have revised in the manuscript. **(Methods Materials and methods section, page 5, lines 128-129)**.

**The revised paragraph now reads as follows:**

“Methods Materials and methods

Participants and study design

…Survey data from 2011-2018 were combined by identity document‌, resulting in a longitudinal survey of 13,263 data. There were 489 data with positive cognitive impairment at baseline**.** Twenty five with missing data in PA and cognitive impairment data at baseline were excluded, and 1487 with missing data in longitudinal cognitive impairment data were excluded, leaving 11262 data.”

Comment 7: line 145, revise sentence to indicate what is "calculated as follows"? the MET score? if so, state this clearly.

**Response:** We sincerely thank for the valuable comment. We have revised "calculated as follows" to " The MET score was calculated as follows " in the manuscript. **(Methods Materials and methods section, page 6, lines 147-148)**.

**The revised paragraph now reads as follows:**

“Methods Materials and methods

Physical activity

…

**The MET score was calculated as follows**: PAV = 8.0 × duration of vigorous-intensity physical activity (VPA) per week + 4.0 × duration of Moderate-intensity physical activity (MPA) per week + 3.3 × duration of light-intensity physical activity (LPA) per week [28]. …”

Comment 8: line 149, suggest "therefore, we elected to divide the results according into two main categories...."

**Response:** We sincerely thank for the valuable comment. We have revised to "therefore, we elected to divide the results according into two main categories...." in the manuscript. **(Methods Materials and methods section, page 6, lines 152-153)**.

**The revised paragraph now reads as follows:**

“Methods Materials and methods

Physical activity

…

According to IPAQ, Insufficient PA is defined as the PAV less than 600 MET minutes/week [29], **therefore, we elected to divide the results according into two main categories** (0-599 and ≥600) and seven subcategories in terms of MET minutes/week (0-599, 600-1199, 1200 -1799, 1800-2999, 3000-5999, 6000-8999 and ≥9000). …”

Comment 9: line 156: suggest "domains" or "functions" not "abilities"

**Response:** We sincerely thank for the valuable comment. We have revised to " functions." in the manuscript. **(Methods Materials and methods section, page 6, lines 159)**.

**The revised paragraph now reads as follows:**

“Methods Materials and methods

Cognitive impairment

…

Cognitive impairment was assessed based on two cognitive **functions**: episodic memory and executive function. Episodic memory was measured by immediate and delayed recall of words. …”

Comment 10: line 165: suggest 'similar to" rather than "as" (in comparing the TICS and the MMSE)

**Response:** We have revised "as" to "similar to" in the manuscript. Thanks. (**Methods Materials and methods section, page 6, lines 168)**.

**The revised paragraph now reads as follows:**

“Methods Materials and methods

Cognitive impairment

…

The TICS is a reliable and valid screening tool for cognitive impairment in older adults similar to the Mini-Mental State Examination (MMSE). …”

Comment 11: line 176: insert "are" between "(BMI)" and categorized"

**Response:** We have inserted "are" between "(BMI)" and categorized" in the manuscript. Thank you. **(Methods Materials and methods section, page 6, lines 179)**.

**The revised paragraph now reads as follows:**

“Methods Materials and methods

Covariates

…

2) Health status-related variables: body mass index (BMI) **are** categorized as follows: underweight (<18.5), normal weight (18.5 ≤ BMI <25), overweight (25 ≤ BMI <30), and obese (BMI ≥30);. …”

*Reviewer #3: PLOS ONE*

*Ref: PONE-D-25-38651*

*Title: “Independent and joint associations of volume and intensity of physical activity on cognitive impairment among middle-aged and elderly Chinese adults: a national longitudinal study”*

- Thank you for the opportunity to review the manuscript entitled “Independent and joint associations of volume and intensity of physical activity on cognitive impairment among middle-aged and elderly Chinese adults: a national longitudinal study”
- The authors essentially measured physical activity and risk for cognitive impairment. They compared participants in the following manner: Baseline characteristics were compared between groups with insufficient vs. sufficient physical activity (PA), using percentages and chi-square tests for categorical variables. They then did a risk analysis using Cox proportional hazards models. Hazard ratios (HRs) were calculated using 95% confidence intervals to assess the association between PA volume/intensity and cognitive impairment risk.
- Model Adjustments were done and presented as model I: Adjusted for age and gender, model II: adding smoking, alcohol use, education, and BMI and a final model III, which Included all study covariates.
- Joint effects were measured /done by means of Restricted Cubic Splines (RCS), which evaluate d the combined effect of moderate-to-vigorous PA (MVPA) and light PA (LPA) on cognitive impairment risk. Participants were the grouped based on MVPA and LPA proportions.
- The subgroup analyses were then conducted separately by: Age (<65 vs. ≥65 years), Gender (male vs. female) and residence (rural vs. urban).
- Statistical analyses were performed using STATA 14 and R, with significance set at p < 0.05.
- Based on this, it seems regardless of how much total physical activity someone does, the mix of intensity matters, with balanced activity — with moderate MVPA (25–50%) and high LPA (50–75%) — linked to the lowest risk of cognitive impairment. Low proportions of either MVPA or LPA are associated with higher risk, even if overall activity levels are high. I suggest the following be highlighted clearly: the importance of diversifying activity intensity, not just increasing total volume.
- Presentation of the results is sound, with the forest plot visually supporting the quantitative relationships described in the text. Both indicate that balanced physical activity — with moderate MVPA and higher LPA — is associated with lower cognitive impairment risk, regardless of total activity volume.
- Regardless of the above summary, in general, I found the manuscript of good quality (some comments for minor edits made re grammar, see attached) and I commend the authors for their original contribution to a very important and relevant area of study in neuropsychiatry. In addition, the cross-cutting issues for exercise science disciplines provides additional value.

The longitudinal nature of the study, as well as big sample size add further add merit.

**Response:** We sincerely thank the reviewers for their insightful and constructive comments, which have significantly improved the quality of our manuscript. We have carefully addressed each point raised, as detailed below. Below, we provide point-by-point responses to their feedback.

A few things for noting:

Comment 1: I suggest that the term “cognitive impairment” be clearly commented on right at the outset. It must be noted that cognitive decline with aging process may be mild, moderate or severe, with classifications for neurodegenerative disorders such as Dementia are generally delineated into minor or major neurocognitive disorders of which Alzheimer’s dementia is an example. From the outset, it would be best to define what exactly this study is examining- cognitive impairment or dementia? In line 87 it appears as if the study refers to neurodegenerative disorders in general.

**Response:** We sincerely thank the reviewer for the valuable suggestion. We have added define cognitive impairment in the context of the study in the introduction. In addition, we have deleted the study appears to examine neurodegenerative disorders in line 87. **(Introduction section, page 3, lines 65-67)**.

**The revised paragraph now reads as follows:**

“Introduction

Cognitive impairment (CI) refers to a significant decline or dysfunction in memory, learning, attention, language, executive function, and other cognitive domains [1], ^…”^

**The relevant certificate involved is listed as follows:**

1.	Petersen, R.C., et al., Mild cognitive impairment: ten years later. Arch Neurol, 2009. 66(12): p. 1447-55.

Comment 2: In the methodology, please indicate clearly (perhaps add to the flow chart on page 5) when exactly were the physical activity measures undertaken (i.e. when was the IPAQ administered?)

**Response:** Thanks for your valuable advice. We have revised in the manuscript. Thank you. **(Methods Materials and methods section, page 6, lines 139)**.

**The revised paragraph now reads as follows:**

“Methods Materials and methods

Physical activity

…

Physical activity was measured by the International Physical Activity Questionnaire (IPAQ). **In each of the four surveys from 2011** to 2018, participants were specifically questioned regarding the duration, frequency (measured in days), and intensity (whether vigorous, moderate, or mild) of physical activities they regularly undertook for at least 10 minutes during a typical week. Based on other scholars’ method [27], we transformed the time ranges by taking the middle value. …”

We appreciate this thoughtful comment, which helps to clarify the conceptual scope of our current analysis and offers important directions for future empirical refinement.
